# Clinical Performance of Implant Crown Retained Removable Partial Dentures for Mandibular Edentulism—A Retrospective Study

**DOI:** 10.3390/jcm10102170

**Published:** 2021-05-18

**Authors:** Soo-Yeon Yoo, Seong-Kyun Kim, Seong-Joo Heo, Jai-Young Koak, Hye-Rin Jeon

**Affiliations:** 1 Department of Prosthodontics and Dental Research Institute, Seoul National University Dental Hospital, School of Dentistry, Seoul National University, 101 Daehak-ro, Jongno-gu, Seoul 03080, Korea; sy0502@snu.ac.kr (S.-Y.Y.); 0504heo@snu.ac.kr (S.-J.H.); young21c@snu.ac.kr (J.-Y.K.); 2 Department of Computer Science, Columbia University, New York, NY 10027, USA; hj2589@columbia.edu

**Keywords:** implant-crown-retained removable partial dentures (IC-RPD), implant overdenture (IOD), survival rate, marginal bone loss (MBL), patient reported outcome measures (PROMs), prosthetic complication

## Abstract

The studies on implant-crown-retained removable partial dentures (IC-RPDs) for edentulism are scarce. The purpose of this study was to evaluate survival rates and marginal bone loss (MBL) of IC-RPDs compared to implant overdentures (IODs) in mandibular edentulism. Variables that influenced survival and marginal bone loss (MBL) of implants in both treatment modalities were analyzed and the functional/esthetic satisfaction of patients as well as prosthetic complications were also observed. Eighteen IC-RPDs with a total of 60 implant-supported survey crowns and 24 IODs with a total 94 implants retained with magnet attachments were observed. After a median observation period of 46.6 months (up to 149 months), we observed 98.3% implant survival rates for IC-RPDs and 92.5% for IODs. Kaplan–Meier survival curves based on the treatment modality showed that, at 96 months, cumulative survival rates were 98.3% in IC-RPD and 83.1% in IOD. For implant survival rates, no statistical differences were observed according to age, sex, opposing dentition, or implant positions (*p* = 0.515, 0.666, 0.201, 0.749, respectively). The implant MBL measurements for IC-RPD and IOD groups at the final recall check were 0.93 ± 1.22 mm and 2.12 ± 2.09 mm, respectively. Additionally, there were no significant differences between groups (*p =* 0.554). The implants with peri-implantitis at year 1 showed significantly higher MBL at final check-up (*p* < 0.001). The MBL of implants showed significant differences based on age (*p* = 0.008) and opposing dentition (*p* = 0.003). No significant differences of implant MBL were observed for the position of placed implants (*p* = 0.621) or sex (*p* = 0.666). Patient-reported outcome measures (PROMs) on functional and esthetic satisfaction were significantly improved after IC-RPD or IOD treatment (*p* < 0.001). The most frequent prosthetic complication of IC-RPD was clasp loosening, while for IOD group, it was attachment dislodgement. Within the limitations of this retrospective study, we concluded that IC-RPDs could be considered as a viable treatment option for edentulous patients who need few fixed abutments for satisfaction.

## 1. Introduction

The continuing complaints of patients with completely edentulous mandibles often pertain to the difficulty of adapting to a removable prosthesis, due to the reduced residual ridge and lack of soft tissue. Common clinical problems with mandibular complete dentures (CDs) include lack of retention and stability in addition to discomfort. For these edentulous patients, implants for implant overlay overdentures (IODs) can be introduced to improve retention and stability. For mandibular edentulism, the fixed prostheses can also be considered however, the fixed prostheses in full-arch rehabilitation should be supported by six to nine implants, which takes a long time and has high costs due to extensive surgeries [1]. Therefore, patients with anatomical and socio-economical limitations have a tendency to select IOD as a routine treatment modality instead of full fixed restoration to avoid additional surgeries and reduce costs. 

IODs are divided into IODs with splinted-type attachments or solitary-type attachments. The bar/clip attachment is a common splinted attachment that compensates for the stress on supporting tissue by transmitting force to the implant and allowing rotation of the prosthesis [2]. The solitary attachments include ball, locator or magnet types; these are connected to implants individually and provide retention through mechanical engagement of male and female parts. 

According to the McGill and York Consensus Statements, regardless of the type of attachment system such as bar/clip, ball and magnet, etc., patients were significantly more satisfied with two-implant ODs than with CDs. Therefore, they suggested the IOD as a more effective and minimal treatment option for the completely edentulous mandible compared to CDs [3,4]. IODs also present the following benefits compared to CDs: (1) better chewing ability; (2) better fit and retention; and (3) improved function and quality of life [5]. 

Previous studies reported that masticatory force exerted on a mandibular IOD is less than natural teeth or implant-supported fixed prostheses [6,7,8]. However, in IODs, horizontal stress is delivered, which is potentially more harmful to the implant and its surrounding tissues than vertical stress [7,9]. Thus, the attachment must provide an optimum stress distribution around the implants to minimize the stress transmitted to the implants and bone. In vitro research and finite element analysis of ODs in the mandible showed that stress and strain around the implant were greatly affected by the implant design, and less stress was generated around implants that were not splinted [10,11,12,13,14]. As a result, to give implants less burden, solitary implants in ODs could be sufficient. Furthermore, the previous study suggested that the attachment system did not influence the success rate of the implants, and other factors such as bone quality, bone quantity, and arch morphology would play far more important roles in implant survival rates [15]. Thus, in this study we set aside which kind of attachments were used for IOD and rather focusing on anatomical conditions more than the attachment systems. 

Recent studies reported that anterior positioning of implants might prevent dislodgment of dentures vertically and horizontally in a removable partial denture (RPD) with implant stops [16,17]. Del’Arco Pignatta Cunha et al. reported that, when comparing the force applied to the abutment, the force distribution was more favorable as the location of the implant moved from the last molar area to the premolar area [16]. Furthermore, according to case reports and long-term clinical studies, additional implants in the front area of the edentulous ridge in RPDs showed satisfying results [18,19,20]. Those previous studies used implants as attachments underneath removable prostheses, however Tarnow et al. did a clinical trial to fabricate implant-supported crowns to use as a partial denture abutment in RPD [21]. Considering the concept of combining RPDs with implant crowns, we designed a new prosthetic modality: the implant-crown-retained removable partial denture (IC-RPD) for mandibular edentulism. To compensate for anatomical limitations due to the position of the alveolar nerve and to reduce treatment cost, furthermore for their satisfaction of fixed prosthesis, patients received fewer implants in the anterior region. In those cases, implant surveyed crowns or bridges were fabricated and RPDs with retentive clasps, rests, and proximal plates were delivered. 

Implant survival rates and marginal bone loss (MBL) around implants are important factors to decide treatment plans related to implants. Usually survival rates for fixed implants in edentulous patients are reported in the range of 92.1% [22] to 95.6% [23]. Kang et al. showed that the survival rate of implants in IC-RPDs for partially edentulous mandible was 93.1% [24]. The 5-year prospective randomized study by Gotfredsen and Holm showed a success rate of 100% in IODs [25]. Overall, implant survival rates have been shown to range from 92.1 to 100% regardless of the treatment modality in previous studies. For MBL, the systematic review of Zimmermann et al. showed the 1 year MBL of fixed restorations was ranged from 0.05 ±0.67 to 1.37 ± 0.5 mm, while the 1 year MBL for mandibular removable restorations ranged from 0.13 ± 0.35 mm to 1.03 ± 0.65 mm [26]. The other recent study reported that fixed and removable implant-supported prostheses showed similar long-term MBL [27]. The IC-RPD of this study is a combination of fixed implants and removable prostheses; therefore, we need to figure out MBL of IC-RPD.

While the studies on survival rates and implant MBL of IC-RPD in mandibular edentulism are very scarce, to the best of our knowledge, no long-term data have been collected for IC-RPDs for fully mandibular edentulous patients. Clinical studies on IC-RPDs have not been performed as often compared to the studies on IOD for fully mandibular edentulism, possibly [28] because the IOD has been a routine treatment modality. However, some patients needed at least few fixed prostheses to relieve their frustration of edentulism and for satisfaction like they had few natural teeth with RPD, in those cases IC-RPDs were delivered. 

The systematic review of Yao et al. found there were heterogeneous results on PROMs of IOD compared to full fixed implant prostheses; however, any type of implant prostheses increased satisfaction after treatment [29]. There is scientific evidence that mandibular IOD could provide predictable results with good retention and high satisfaction [30]. Satisfaction of patients is one of main reason for oral restoration. Therefore, for new treatment modality for edentulism, we need to check PROMs. Goodacre et al. reported the loosening of the overdenture retentive mechanism was most common incidence of implant related complications [31]. However, there was no study related to mechanical complications of IC-RPD in mandibular edentulism.

The aim of this study was to verify survival rates and MBL of IC-RPD compared to conventionally considered IOD in mandibular edentulism. Variables that influenced survival and MBL of implants, such as first year pathologic condition, age, sex, opposing dentition, and location of implant placement (anterior vs. posterior), were analyzed in both treatment modalities. The functional/aesthetic satisfaction of patients as well as prosthetic complications were also observed to evaluate IC-RPDs. The null hypothesis was that regarding survival rates and MBL of implants in IC-RPD, no differences compared to IOD were found; additionally, there were no statistical differences between PROMs and prosthetic complications in groups (IC-RPD vs. IOD). 

## 2. Materials and Methods

### 2.1. Study Protocol and Eligibility Criteria 

Our study sample was drawn from 49 edentulous patients who were treated with IC-RPD or IOD for mandibular edentulism between January 2012 and July 2020 at Seoul National University Dental Hospital and S leader dental clinic in South Korea. This study was authorized by the Institutional Review Board of Seoul National University Graduate school of Dentistry (No. S-D20200040). All patients included in this study were treated by surgical or prosthodontic specialists and underwent periodic recall checks.

Of the 49 patients, we ultimately included 42 (20 men, 22 women) patients and bone level internal type 154 implants. Patients with systemic diseases (e.g., diabetes, osteoporosis) affecting implant prognosis and patients who had any conditions that contraindicated denture recall were all excluded. The study sample was divided into two groups: edentulous patients who wore IODs (i.e., implant overlay complete dentures) and edentulous patients who wore IC-RPDs with splinted implant surveyed crowns.

According to our clinical charts, inclusion criteria for the placements of 154 implants were: (1) adequate bone to accommodate two to four implants over the arch; (2) no severe systemic problems, fair health, and the ability to undergo a surgical procedure with local anesthesia; (3) no drug or alcohol abuse (a smoking cessation program was provided to smokers before treatment); and (4) no unrealistic demands regarding treatment outcome. 

### 2.2. Data Collection and Analysis

**Figure 1:**
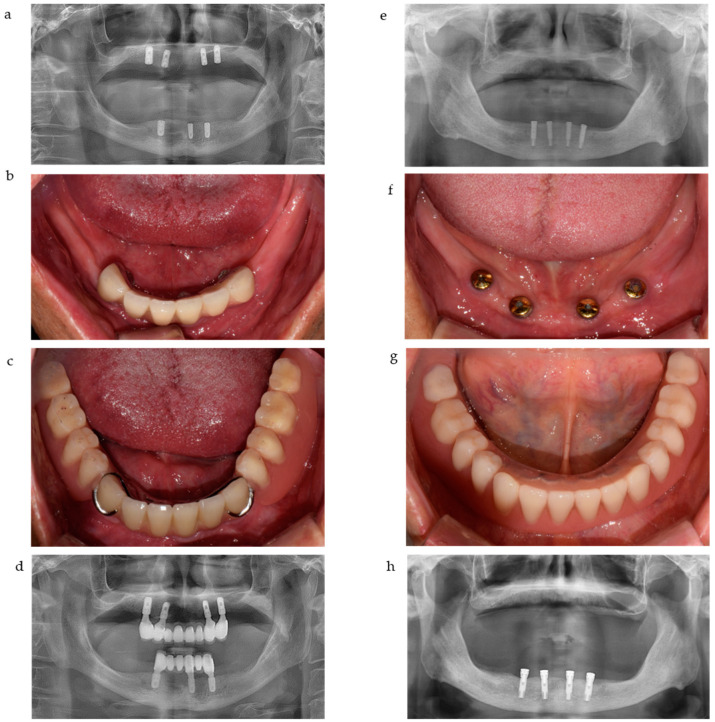
The representative cases of IC-RPD and IOD in this study. (**a**) Implants were placed in anterior positions due to anatomical limitations. (**b**) Intraoral view of IC-RPD case. (**c**) Delivery of IC-RPD. (**d**) Panoramic radiograph of IC-RPD case. (**e**) Four implants were placed in anterior positions due to anatomical limitations. (**f**) Intraoral view of IOD case. (**g**) Delivery of IOD. (**h**) Panoramic radiograph of IOD case.

All clinical and radiographic assessments were performed randomly on a total of 154 implants. All implants in IC-RPDs were placed in anterior (canine or premolar position) areas. Thus, Class I IC-RPDs (or Class I with a modification) were delivered to 18 patients (Table 1). For IOD cases, symmetrically distributed (within the limitations allowed by anatomical conditions) implants were attached with magnets to the bottom of the IOD as shown in Figure 1.

All 154 implants in this study were of a regular internal type (Table 2); 128 had diameters of 4–4.5 mm (with lengths of 10 mm or 11.5 mm) and 26 had diameters of 4.8–5 mm (with lengths of 8.5 mm or 10 mm). The total 18 IC-RPDs were assisted by 60 implant-supported porcelain fused metal (PFM) surveyed crowns and 24 IODs were held by 94 solitary magnets (Table 2). The follow-up period in this study ranged from 12 to 149 months (mean 46.6 months).

At delivery of prostheses, intraoral evaluation of the occlusion was performed and maintenance instructions as well as oral and written presentations of each patient’s recall schedule were noted. Follow-up on all patients was conducted annually for at least 1 year to 13 years. The following evaluations were made during follow-up: (1) implant survival; (2) radiographs of implant MBL; (3) PROMs at 6-month recall check; and (4) prosthetic complications.

The main outcome in this study was cumulative implant survival rate. The implant survival criteria we used followed the Pisa consensus statement of the ICOI Conference in 2007 [32]. Implants were considered to have survived if the implant and its superstructure were functioning normally at the final observation. 

Peri-implant bone resorption was evaluated with annual intraoral radiographs, using digitized panoramic and periapical radiographs. To eliminate bias, all radiographic data were collected and categorized by order of chart number regardless of treatment modalities and evaluations were randomly conducted by a single examiner (SYY) according to the same criteria twice. The Intraclass Correlation Coefficient (ICC) value is the reliability calculated by the raters’ measurements. The ICC means reproducibility if the test is repeated several times. Therefore, for reliability of measurement in this study, ICC values were statistically analyzed. Radiographs taken during the final recall visit were used to determine the peri-implant bone level as the distance between the platform of the implant and the level of the adjacent osseous crest on the mesial and distal aspects, respectively. Based on the actual length of the implants, the actual bone level was calculated by a proportional equation [33]. We defined MBL as the differences between mean value of bone resorption in the mesial and distal aspects at final visit and implant delivery. 

In this study, we observed MBL around implants based on multiple variables, such as first year pathologic condition, age, sex, opposing dentition, and location of implant placement. The current guidelines for the diagnosis of peri-implantitis were defined by the 2017 World Workshop on the Classification of Periodontal and Peri-implant Diseases and Conditions [34]: (1) presence of bleeding on probing (BOP) and/or suppuration; (2) increased probing depth (PD); and (3) presence of detectable bone loss exceeding measurement error (mean 0.5 mm) with radiographically observed first year pathologic condition. However, there are various opinions to define peri-implantitis. Ramanauskaite et al. suggested the rationale for diagnosis of peri-implantitis [35], and many authors have followed the consensus from the First European Workshop suggesting the criteria of implant success as MBL of less than 1.5 mm during the first year after the insertion of the prosthesis and thereafter less than 0.2 mm annual bone loss [36]. Other authors have reported that changes ≥2 mm at any time point during or after the first year should be considered as pathologic (i.e., peri-implantitis) [37,38,39]. In this study, we diagnosed peri-implantitis as having MBL that was superior to 1.5 mm with the presence of increased PD, BOP and/or suppuration [40]. 

Patient quality of life and satisfaction are the main considerations when choosing treatment modality [41]. In this study, we examined PROMs after IC-RPD or IOD treatment according to visual analog scales (VAS) of 1 to 5, in which 1 was the least favorable. Our questionnaires asked patients to: (1) rate before and after esthetic satisfaction with the prosthesis procedure; and to (2) rate before and after functional satisfaction with the prosthesis procedure. Satisfaction levels were recorded after IC-RPD or IOD prosthesis insertion (usually at the 6-month check-up). 

Finally, we collected all data from clinical charts regarding occlusion and technical complications after prosthesis delivery. All chart records were reviewed to identify complications associated with RPD or implant surveyed crowns in IC-RPDs, and complete denture (CD) or implant attachments in IODs. Prosthetic complications were classified into 5 categories: (1) denture: fractures or deformations of the denture components followed by repair or fabrication of new dentures; (2) implant: screw loosening or fractures; (3) implant surveyed crowns in IC-RPD: dislodgement of prostheses or veneer porcelain fracture in PFM; (4) magnet attachments in IOD: mobility, dislodgement or loss; and (5) tissue: sore spots or crestal bone resorption due to denture base. 

### 2.3. Statistical Methods

All data were evaluated using the statistical package SPSS version 23 (SPSS Inc., Chicago, IL, USA). In order to analyze the cumulative survival rate (CSR) of implants, the Kaplan–Meier method was used with a log rank (Mantel–Cox) test to compare variables. The time interval criterion for implant failure and implant MBL was defined as the time difference between delivery date of the prosthesis and complication occurrence date and/or observation end date. For analysis of final bone loss, we adjusted values by time using mixed analysis due to the differences of observation period. 

We used the Kruskal–Wallis test to configure the differences of survival rates and MBL of implants according to variables such as first year pathologic condition, age, sex, location of implant placement and opposing dentitions; additionally, we ran a Mann–Whitney test with the results. To confirm reliability of measurement on implant MBL, ICC was also analyzed at 95% confidence interval in this study.

In addition, we used the Wilcoxon signed rank test to detect significant functional or esthetic improvements after treatment, and also initially applied the Kruskal–Wallis test to determine differences in PROM variables. With the results derived, we made final comparisons using the Mann–Whitney test. 

## 3. Results

### 3.1. Implant Survival Analysis

During the observation period, 8 of 154 implants failed; therefore, the total survival rate of implants was 94.8%. Table 3 depicts the specific information of the eight failed implants of three patients (patient A from the IC-RPD group, patient B and C from the IOD group). In comparison of survival rates according to treatment modalities, one implant as a surveyed crown in IC-RPD and seven implants in IOD failed, resulting in survival rates of 98.3% for implants in IC-RPD and 92.6% for implants in IOD. There were no statistically significant differences in failure rates by treatment modality (*p =* 0.116). One failed implant from the IC-RPD group showed the smallest survival period (18 months) and it was occluded to natural abutment teeth with maxillary RPD. This implant (44i) of patient A failed by mobility with long radiolucency around implant threads and pain. The other two implants (33i,34i) of patient A still survived at final check-up (for 33 months). 

Seven failed implants with the IODs were from only two patients (patient B and C in Table 3), whose implants were nearly all subsequently removed. Four implants of patient B were previously supported by full mandibular fixed prostheses, but constituent failures led to use of IODs with four solitary magnet-attached implants beneath the denture base. Additionally, these four implants were also all removed, 67–75 months after implant placement. Another three failed implants were from patient C whose old dentures showed severe attrition, resulting in collapsed vertical dimension. Fortunately, an implant (46i) of patient C survived at final check-up (for 47 months). Both patient B and C had bruxism before they lost their natural teeth, along with unfavorable habits such as chewing foods with lateral movements, according to their clinical charts. 

**Figure 2:**
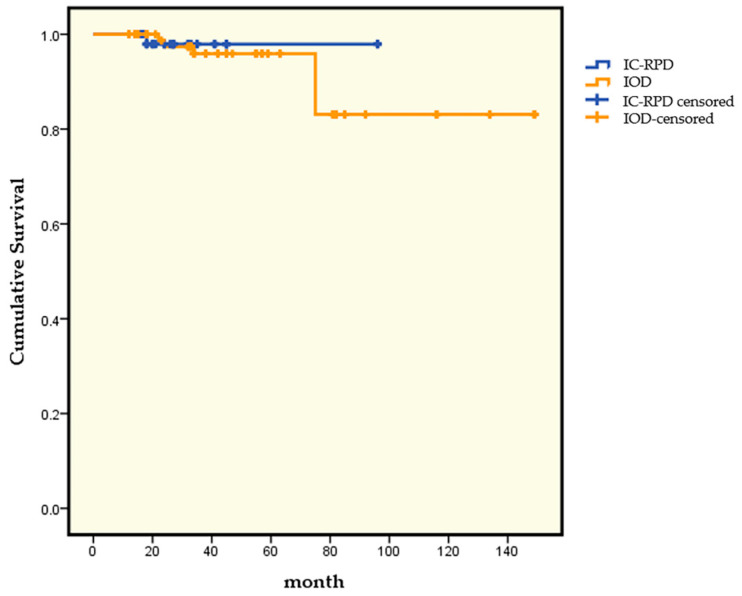
Kaplan–Meier survival curve according to treatment modality (IC-RPD vs. IOD).

Kaplan–Meier survival curves based on the treatment modality are illustrated in Figure 2. At 75 months, CSRs were 98.3% in IC-RPD and 95.9% in IOD, whereas at 96 months, CSRs were 98.3% in IC-RPD and 83.1% in IOD. 

Table 4 shows first year pathologic condition affected survival rate of implants in both groups. There were no significant differences between implant failure according to age or sex (*p* = 0.515, 0.469, respectively. The position of implants (anterior or posterior) also showed no differences in failure rates (*p* = 0.749). The occluding dentitions of patients who had failed implants were either natural tooth or IOD in this study, and there were no statistically significant differences of implant failure according to opposing dentition (*p* = 0.435).

### 3.2. Implant Marginal Bone Loss Analysis

Table 5 shows the analysis of MBL around the implants in IC-RPDs and IODs. The mean MBL in all patients at year 1 was 0.64 mm, while the mean implant MBL at final recall check was 1.65 mm. The implant MBL through time frame shows significant changes by mixed model analysis (*p* < 0.0125; adjusted by Bonferroni correction). However, the average time of final check-up dates (i.e., observation end date) was different: 32.3 months for the IC-RPD group and 64.7 months for the OD group. Therefore, final check-up marginal bone loss was adjusted by time variances. There were no differences found (*p* = 0.544) between groups at final check-up. 

**Figure 3:**
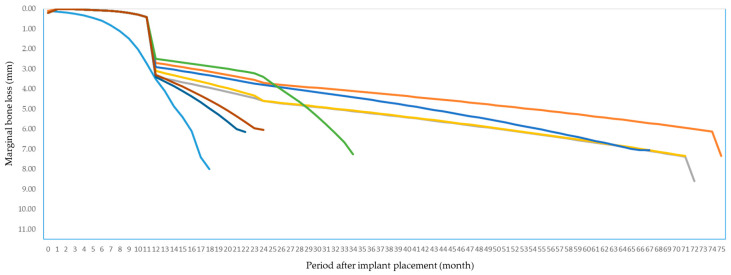
Marginal bone loss (MBL) of 8 failed implants shown in different colors. Most of the failed implants showed early bone loss.

ICC of MBL measurement was 0.99 at year 1 after loading, 0.983 at year 2 after loading and 0.988 at final check-up. All MBL measurements exhibited an excellent reliability, based on the 95% confident interval of the ICC estimation The MBL of failed implants is illustrated in Figure 3. Most of the failed implants showed early bone loss, 1 year after implant placement. 

For both IC-RPD and IOD groups (*p* < 0.001), when peri-implantitis was observed at year 1, the final implant MBL for these groups showed significantly higher bone loss (Table 6). Additionally, age groups were divided into the following 4 groups: under 60, 61–65, 66–70, and above 70 years old. There was a significant difference based on age (*p* = 0.008). Final comparisons of Mann–Whitney test showed that the elderly patients, 66–70 years old age group resulted in less bone loss around implants than younger patients under 60 years old or 61–65 years old group (Table 7). 

No significant difference could be observed for sex (*p* = 0.666) or for the position of Implants (anterior vs. posterior; *p* = 0.621). However, the opposing dentition (the upper dentition in this study) significantly affected implant MBL (*p* = 0.003) in both IC-RPD and IOD groups, as shown in Table 8. Opposing dentition groups were divided by five groups as natural teeth, implants, IOD, RPD and CD. We ran post comparisons of Mann–Whitney test and the result showed when the opposing dentition was an IOD, mandibular implants resulted in highest implant MBL; 2.75 ± 2.22 mm. When the opposing dentition was implant, MBL of mandibular implants showed the smallest value 0.83 ± 0.76 mm. 

### 3.3. PROMs

**Figure 4:**
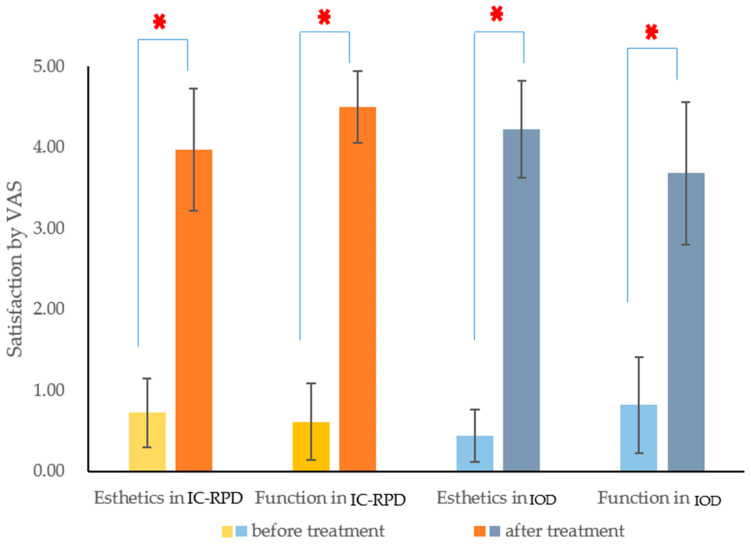
Comparison in satisfaction rate (esthetic and function) by VAS before vs. after prosthetic treatment. The Wilcoxon-Signed Ranks test showed there were significant differences before and after treatment in both esthetics and function regardless of the treatment modalities (*p* < 0.05). Asterisk (*) indicated statistical differences (*p* < 0.001).

In both IC-RPD and IOD groups, the satisfaction of patients was improved significantly (*p* < 0.001) after the delivery of new prostheses according to the Wilcoxon signed rank test (Figure 4).

**Figure 5:**
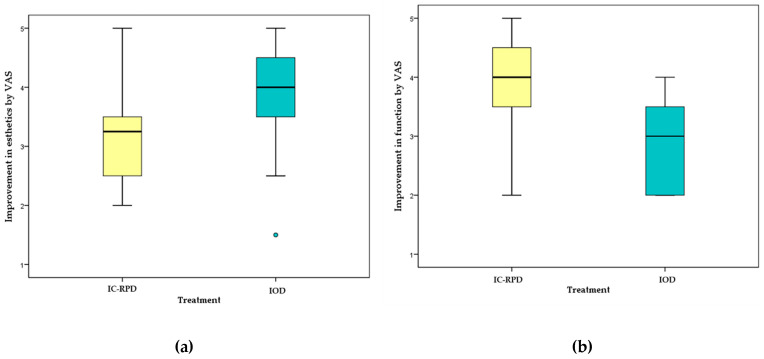
Esthetic and Functional improvement by VAS according to treatment modality (IC-RPD vs. IOD). (**a**) The OD group showed higher measured values by VAS in esthetic improvement (*p* = 0.024). (**b**) The IC-RPD group showed a significantly higher value in functional improvement (*p* < 0.001). Green dot indicated only one patient with VAS value of 1.5 after delivery of IOD.

Considering the function as mastication ability, there were significant differences between groups (*p* < 0.001). The value of VAS on masticatory function was significantly higher in the IC-RPD group (Figure 5). For the esthetic appearance, patients who wore IC-RPDs in the mandible were not as satisfied as those who wore ODs. The value of VAS on esthetic appearance improvement was significantly higher in the OD group (*p* = 0.024). 

### 3.4. Prosthetic Complications 

The mechanical complications in both treatment modalities were divided into five categories and analyzed (Table 9 and Table 10). After the delivery of prosthesis, the most common mechanical complication in IC-RPD was clasp loosening (44% of complication incidences) and this was followed by sore spot under denture base (37%). 

For the IODs, mobility or dislodgement of magnet attachment was the most frequent complication (29.2% of complication incidences), and sore spots under the denture base (26.8%) also occurred often. Crestal bone loss under the denture base was also often observed 20.7% in the IOD group. Four cases of fracture of denture base around implant attachment and four cases of artificial tooth fracture were reported in IOD group. All complications were resolved by repairing or changing the components. 

Overall, the occurrence date of average mechanical complication was much earlier in the IC-RPD group while sore spot under denture base or crestal bone resorption occurred later in the IC-RPD group than the IOD group.

## 4. Discussion

The null hypothesis that survival rates and MBL of implants in IC-RPD were found no differences compared to IOD was accepted. However, null hypothesis that there were no statistical differences between PROMs and prosthetic complications in groups (IC-RPD vs. IOD) were disapproved. 

The results showed the implant survival rates were 98.3% for the IC-RPD group and 92.5% for the IOD group. There was no statistically significant difference in survival rate by treatment modality (*p* = 0.116). However, there was a limitation of different observation period between groups (IC-RPD vs. IOD). Therefore, to compensate our results, we applied Kaplan–Meier survival curve analysis in which observation period is not important. According to Kaplan–Meier survival curve analysis, at month 75, the implant CSR was 98.3% in IC-RPDs and 95.9% in IODs. However, at 96 months, the implant CSR was still 98.3% in IC-RPDs but the implant CSR of IODs decreased to 83.1%. The IOD has been reported as a clinically acceptable treatment modality [42] and used as a routine treatment option for mandibular edentulism. Considering CSR of IOD, CSR of IC-RPD showed better results in this study however, we cannot conclude IC-RPD is an alternative treatment modality to IOD and need further long term cross-sectional studies. 

In the present study, one failed implant in the IC-RPD group occluded with natural tooth and the seven failed implants in the IOD group occluded with the IOD (Table 3). All of these patients had a history of bruxism or clenching before treatment. The occluding dentitions of these patients showed severe attrition before treatment and newly fabricated IC-RPDs or IODs also showed uncontrolled severe attrition within 1 year. We assumed that excessive loading due to the clenching or abnormal mandibular movement after treatment caused the failure.

Linear mixed model was applied to analyze bone loss at defined observation time (at delivery, at 1 year and 2 years after loading, at final check-up). Additionally, to overcome different observation periods at final check-up between groups, we added individual period variance for MBL mixed analysis. In our study, the mean MBL of implants for the IC-RPD group at the final recall check (up to 75 months) was 0.93 ± 1.22 mm and for the IOD group (up to 149 months) was 2.12 ± 2.09 mm. The total MBL of 145 implants in all patients was 1.65 ± 1.89 mm in our study. These results are within a clinically normal range of bone loss. Additionally, not like MBL of at year 1 and year 2, we did not find any significant differences on implant MBL between treatment modalities (IC-RPD vs. IOD) at final check-up (Table 2). There was a difference only based on time (*p* = 0.028). 

According to the study of Bae at el., MBL of implants in IC-RPDs was 1.44 ± 0.57 mm and 1.99 ± 0.7 mm for implants in IODs after a 6-month examination [43]. This result corresponds with our study; however, we examined for a longer period of up to 149 months. Our study did not show a higher MBL of implants in IC-RPDs or IODs compared to fixed implants due to unfavorable horizontal forces caused by the retentive part of removable prostheses, either.

A previous study reported that the MBL around implants supporting mandibular IODs was not affected by attachment type, age, or sex [44]. However, our study showed a significant difference in the MBL of implants based on age (under 65 years old vs. above 65 years old). Furthermore, pathologic condition (i.e., peri-implantitis) at year 1 affected the MBL of implants; at final check-ups, a more severe MBL was observed in the implant group with peri-implantitis. Therefore, early pathologic changes should be monitored carefully to prevent progressive MBL. 

The patients’ quality of life (QOL) and satisfaction is one of the main considerations in successfully choosing a treatment modality [41]. According to the PROMs in our study, patients treated with IC-RPDs or IODs indicated that there was marked functional and esthetic improvement when we only used 3–4 implants and patients were satisfied with their treatment. In addition, the acceptable survival rates and MBL around implants for the long-term period presented by this study supported that, when there is not enough bone quality or quantity for fixed restoration in fully mandibular edentulous patients, clinicians can recommend IC-RPDs or IODs to their patients. 

The VAS for functional improvement showed a significant difference (*p* < 0.001) between IC-RPDs and IODs: patients with IC-RPDs felt more satisfied with their masticatory ability compared to those with IODs. The reasons behind this result could be that the few fixed crowns might relieve a patient’s frustration caused by tooth loss, and the support and retention provided by implants used as surveyed crowns in the IC-RPDs may help patient mastication. In a previous study on IODs, not only subjective chewing ability but also objectively measured chewing efficiency was improved based on a test diet [45]. In our study, only one patient in the IOD group reported discomfort and a decrease in masticatory force, and we assumed that this was because their IOD was made to salvage failed fixed full implant prostheses. 

For the esthetic aspect, IC-RPD patients were not as satisfied with their appearance as were the IOD patients (*p* = 0.024). We assumed that this was because of the position of the implants. Some implants in the IC-RPDs were placed symmetrically at the canine or premolar position due to anatomical limitations of a narrow anterior ridge and a high alveolar nerve position. When these implants were not connected with an anterior long bridge due to cost, patients were not satisfied with their look because of the prominent clasps at the anterior region and no anterior teeth. 

The most frequent mechanical complication of IC-RPDs was the clasp loosening over time because of the repetitive insertion, but this could be simply resolved by adjustment. However, dislodgements of attachments were the most frequent prosthetic complications in the IOD group and some patients lost their attachments, thus needing to pay more in maintenance fees. Crestal bone resorption or sore spots underneath the denture base were also observed more frequently in the IOD group with a shorter period compared to IOD group. However, the average overall observation period was much longer in the IOD group; therefore, it is hard to determine frequency on mechanical complication of IOD is higher. 

We used magnet attachments for the IOD group and there were no reported complaints due to lack of retention for at least 1 year; however, after 2 years, resorption of posterior bone sometimes resulted in dislodgement of dentures. The attachments of those patients often wore away because of unfavorable movement, thus we needed to reline the denture base and/or change attachments. Even though there were none of the patient reports about loss of retention or implant screw loosening after delivery of IC-RPDs in this study, the observation period of IC-RPD was relatively short therefore, we cannot conclude IC-RPD showed better results either. 

To fabricate IC-RPDs or IODs in mandibular edentulous patients, clinicians need to consider the number of implants to be placed. The fixed prostheses for full arch rehabilitation need to be supported by at least six to nine implants while IC-RPDs or IODs usually only need two to four implants in the mandible [1]. The 2015 EAO consensus assessed that an implant-supported OD using two implants was a cost-effective treatment [46]. The McGill and York Consensus Statements also evaluated OD with two implants as an effective treatment option [3,4]. When a minimal number 1–2 of implants are placed, the implant attachments in IODs are subjected to increased stress and wear [15], compared to a slightly better outcome shown with four implants in IODs [47]. Therefore, considering anatomical condition and cost-effectiveness, two to four implants for IODs and IC-RPDs would be sufficient.

Implant location should be decided carefully, considering dislodgement force and possible future options to change to fixed implant prostheses. Ortiz-Puigpelat et al. concluded that placing an implant in the position of the first molar improves the biomechanical behavior of implant-asssisted RPD [48]. However, an inadequate posterior ridge dimension could restrict implant placement to a more anterior location [20]. According to a study by Cunha et al., as the location of the implant moved from the last molar area to the premolar area, the force distribution observed became more favorable [16]. There are also many case reports and long-term clinical studies that placed implants in the anterior region of the edentulous area and showed satisfying results [18,19,20]. In short, combinations of anterior implants used as surveyed crowns and distal extension RPDs as well as IODs with anteriorly positioned implants could be clinically acceptable options, especially for patients with a severely absorptive ridge in the posterior area. 

In one case of this study, two implants for the IC-RPD group were placed in the lateral incisal teeth position in the mandible and not in canine or premolar positions due to the severely narrowed alveolar bone and high-positioned alveolar nerve. At 4 months, we relined the denture base and afterwards the term of relining was too short compared to other IC-RPDs. We assumed that a too anterior position of implants caused extreme combination syndrome, which resulted in a too long saddle area of IC-RPD which aggravated the resorption of the posterior ridge. Furthermore, the inclination of the posterior ridge was ascending in this case, which Cunha mentioned in his study could maximize the lever arm and therefore exert an unfavorable force on the posterior ridge in the use of free-end removable partial dentures [49]. Therefore, when clinicians chose IC-RPDs for fully mandibular edentulous patients, anteriorly-placed implants should be acceptable; however, there is still a need to examine the position closely to ensure the best force distribution within the range allowed by anatomical limitations. 

Implant placements for IC-PRDs and IODs are difficult. For IC-RPDs, clinicians choose placement based on location of restoring abutment teeth while, for IODs, clinicians have to focus on the divergence of implant axis for attachments. Thus, before placement of implants for IC-RPDs or IODs, accurate plans with a study model need to be conducted.

Consequently, combination of anteriorly placed implants as surveyed crowns and distal extension RPD could act as a viable treatment modality for a patient who want fixed prostheses at lower cost for satisfaction. For conventional cases of mandibular edentulism, IOD is sufficient however if patients do not want to collapse their height of occlusion without removable prostheses and look like they have remnant teeth with anterior implant abutments, IC-RPD could be considered. In this study, the mean observation period in the IOD group (64.7 months) was much longer than in the IC-RPD group (32.3 months), therefore further cross-sectional and prospective studies with longer observation time are necessary. Additionally, due to the limitation of retrospective study, between two groups, sample sizes were different. Even though post hoc calculation of effect sample size through G power software (G power 3.1; significant α level was 0.05) showed the power was 0.83, in further studies, there is a possibility to show different results with ours.

## 5. Conclusions

The survival rates of implants for IC-RPD were 98.3%, while implants for IOD were 92.6%. There was a no statistically significant difference by treatment modality (*p* = 0.116). The implant MBL for IC-RPD group was 0.93 ± 1.22 mm, while for IOD group, it was 2.12 ± 2.09 mm at final check-up. There was no statistical difference either (*p* = 0.544). The functional and esthetic satisfaction was significantly improved in both groups after IC-RPD or IOD treatment (*p* < 0.001). The most frequent prosthetic complication of IC-RPD was the clasp loosening, while for IOD group, it was the attachment dislodgement. The absolute number of prosthetic complication incidences were higher in IOD; however, there are limitations that the observation time and sample sizes of two groups are different. Under the limitations of this retrospective study, it was concluded that both IC-RPDs with implant crown and IODs with solitary attachments would be appropriate treatment options for mandibular edentulism. 

## Figures and Tables

**Table 1:** Kennedy–Applegate classification in the implant-crown-retained removable partial denture (IC-RPD) group.

IC-RPD Kennedy’s Classification	w/ or w/out Modification	Implant Position	IC-RPD Number
Class I	no modification	anterior	6
	with a modification	anterior or premolar	12

**Table 2:** The number of implants for IC-RPD and IOD and related information.

Treatment Modality	Implant Connection Type	Implant Manufacturer	Implant Diameter	Total
IC-RPD (*n* = 18)	Internal type	Osstem	Regular (4,4.5 mm)	38
			Regular (5 mm)	6
		Dentium	Regular (4.3 mm)	12
			Regular (4.8 mm)	4
Overdenture (*n* = 24)	Internal type	Osstem	Regular (4,4.5 mm)	50
			Regular (5 mm)	8
		Dentium	Regular (4.3 mm)	28
			Regular (4.8 mm)	8

**Table 3:** The specific information of the 8 failed implants.

Condition	Patients Who had Failed Implants		
	Patient A	Patient B	Patient C
Patients Age/Sex	69/Male	75/Female	63/Male
Treatment modality	IC-RPD	Implant overdenture (IOD)	
Location of implant	44	36,33,43,46	36,33,43
Diameter/length of implant (mm)	4.0/10	Ant. (4.3/10)	Ant. (4.0/10)
		Post. (4.8/10)	Post. (4.0/10)
Survival periods (months)	18	75/72/71/67	34/22/24
Opposing dentition	Natural tooth #15–14 + RPD	IOD	IOD
Reason of failure	Mobility, pain	Severe bone loss, exudate	Pain, exudate

**Table 4:** Survival rate of implants and *p*-value according to variables.

Condition		No. of Implants	Failed Implants	Survival Rate (%)	*p*-Value
**First year pathologic condition**	With peri-implantitis	21	7	66.6	<0.001
	Without peri-implantitis	132	0	100	
Age	under 60	4	0	100	0.515
	61–65	30	3	90	
	66–70	42	1	97.6	
	above 70	78	4	94.8	
Sex	Male	77	4	94.8	0.469
	Female	77	4	94.8	
Location of implant placed	Anterior position (incisor or canine)	84	4	95.2	0.749
	Posterior position (premolar or first molar)	70	4	94.3	

**Table 5:** MBL of implants in IC-RPDs and IODs at year 1 and at end date of observation.

	IC-RPD (*n* = 60)	OD (*n* = 94)	Total (*n* = 154)	*p*-Value
At year 1	0.1 ± 0.95 mm	0.86 ± 0.92 mm	0.64 ± 0.82 mm	<0.001
At year 2	0.59 ± 0.17 mm	1.13 ±0.11	0.95 ± 1.05	0.004
At end date of observation	0.93 ± 1.22 mm	2.12 ± 2.09 mm	1.65 ± 1.89 mm	0.544

**Table 6:** MBL of implants in IC-RPDs and IODs based on multiple variables.

Condition		No. of Implants	Bone Loss (mm)	*p*-Value
First year pathologic condition	With peri-implantitis	21	2.62 ± 2.25	<0.001
	Without peri-implantitis	133	0.53 ± 0.77	
Age	under 60	4	2.65 ± 0.27	0.008
	61–65	30	2.15 ± 1.90	
	66–70	42	1.06 ± 1.43	
	above 70	78	1.74 ± 2.07	
Sex	Male	77	1.64 ± 1.78	0.666
	Female	77	1.65 ± 2.01	
Location of implant placed	Anterior	84	1.46 ± 1.47	0.621
	Posterior	70	1.16 ± 2.10	

**Table 7:** *p*-value and MBL by age.

Age	Bone Loss (mm)	*p*-Value
under 60 vs. 61–65	2.65 ± 0.27 vs. 2.14 ± 1.90	0.18
under 60 vs. 66–70	2.65 ± 0.27 vs. 1.06 ± 1.43	0.003 < 0.0083
under 60 vs. above 70	2.65 ± 0.27 vs. 1.73 ± 2.07	0.052
61–65 vs. 66–70	2.14 ± 1.90 vs. 1.06 ± 1.43	0.005 < 0.0083
61–65 vs. above 70	2.14 ± 1.90 vs. 1.73 ± 2.07	0.1
66–70 vs. above 70	1.06 ± 1.43 vs. 1.73 ± 2.07	0.156

**Table 8:** *p*-value and MBL by opposing dentition.

Opposing Dentition	Bone Loss (mm)	*p*-Value
Natural teeth vs. Implants	1.73 ± 1.82 vs. 0.83 ± 0.76	0.107
Natural teeth vs. IOD	1.73 ± 1.82 vs.2.75 ± 2.22	0.07
Natural teeth vs. RPD	1.73 ± 1.82 vs. 1.23 ± 1.14	0.526
Natural teeth vs. CD	1.73 ± 1.82 vs. 1.38 ± 1.99	0.157
Implants vs. RPD	0.83 ± 0.76 vs. 1.23 ± 1.14	0.317
Implants vs. IOD	0.83 ± 0.76 vs. 2.75 ± 2.22	0.001 < 0.005
Implants vs. CD	0.83 ± 0.76 vs.1.38 ± 1.99	0.737
RPD vs. CD	1.23 ± 1.14 vs. 1.38 ± 1.99	0.374
RPD vs. IOD	1.23 ± 1.14 vs. 2.75 ± 2.22	0.007
CD vs. IOD	1.38 ± 1.99 vs.2.75 ± 2.22	0.001 < 0.005

Abbreviation: CD; complete denture.

**Table 9:** Complications in IC-RPDs with implant crowns.

	Prosthetic Complication	Number of Incidences (*n*/%)	Average Time of Complication Occurrence (Months)	Mean of Total Follow up Time (Months)	Remarks
Denture	Fracture of RPD clasp	1/3.7	24	26	Repair (change to wrought wire clasp)
	Fracture of RPD rest	-		-	
	Fracture of artificial teeth	-		-	
	Clasp loosening	12/44	18.5	54.1	Akers’ clasp
Implant	Implant screw loosening	-		-	Retightening
	Implant screw fracture	-		-	Change to new screw
Crown	Dislodgement			-	Re-cementation
	Crown veneer fracture			-	Repair
Tissue	Sore spot around Major connector	2/7.4	2	54.5	Relief
	Denture base sore spot	10/37	19.62	23.7	Relief
	Crestal bone resorption	2/7	60.5	96	Relining

**Table 10:** Complications in IODs with magnet attachments.

	Prosthetic Complication	Number of Incidences (*n*/%)	Average Time of Complication Occurrence (Months)	Mean of Total Follow Up Time (Months)	Remarks
Denture	Fracture of artificial teeth	4/4.8	35	68.75	Repair
	Denture base fracture	4/4.8	36.5	48	Around implant attachment -> Repair
Implant	Keeper screw loosening	3 /3.6	36.6	89	Retightening with 30N torque
	implant screw fracture	-	-	-	Change to new screw
Attachment	Mobility or dislodgement	24/29.2	49	89	Reattachment
	loss of attachment	6/7.3	1.5	25.5	Change to new attachment
Tissue	Sore spot around Major connector	2/2.4	15.3	65.2	Relief
	Denture base sore spot	22/26.8	15.3	65.2	Relief
	Crestal bone resorption	17/20.7	54.4	88.2	Relining

## Data Availability

Data sharing is not applicable.
